# Accuracy of refractive error measurements in children with strabismus comparing cycloplegic autorefractometry to dry monocular Mohindra retinoscopy

**DOI:** 10.1371/journal.pone.0323750

**Published:** 2025-06-02

**Authors:** Anna Przekoracka-Krawczyk, Alicja Brenk-Krakowska, Monika Wojtczak-Kwaśniewska

**Affiliations:** Laboratory of Vision Science and Optometry, Faculty of Physics and Astronomy, Adam Mickiewicz University, Poznan, Poland; University of Hyderabad, INDIA

## Abstract

**Objective:**

Uncorrected or undercorrected refractive errors affect 153 million individuals globally, leading to vision impairment and associated quality of life issues. Accurate measurement of refractive errors in children especially with strabismus is critical for effective management. This study evaluates the agreement between near retinoscopy (Mohindra retinoscopy) and cycloplegic autorefractometry in measuring refractive errors among children diagnosed with strabismus.

**Methods:**

Forty-three strabismic children (18 males, 25 females; ages 3–10 years; mean age 5.5 yo) underwent refractive error evaluations using both methods: Mohindra retinoscopy and cycloplegic autorefractometry. The study assessed spherical component, spherical equivalent, cylinder power, and cylinder vectors J0 and J45. Statistical analyses, including Wilcoxon test Student’s t-test, Bland-Altman analysis, Intraclass Correlation Coefficient (ICC) analysis and correlations (supplementary analysis) evaluated differences, correlations and agreement between the two methods.

**Results:**

The median spherical component of refractive error was almost equal for both methods used (+4.38 D vs. + 4.50, for Mohindra retinoscopy and cycloplegic autorefractometry, respectively; *P* = .54), as well as median spherical refractive equivalent (+4.25 for both methods; *P* = .29). Mean spherical components of refractive errors were nearly identical across both methods, showing Mohindra retinoscopy provided slightly higher hyperopic measurements (0.03 D more positive), whereas spherical equivalent of refractive error showed 0.07 D more positive results for Mohindra retinoscopy than cycloplegic autorefractometry. Median cylinder powers were the same for both methods (-0.50 D; *P* = .25). No differences were also found for J0 (*P* = .91) and J45 vectors (*P* = .88) between Mohindra retinoscopy and cycloplegic autorefractometry.

**Conclusions:**

Near retinoscopy demonstrates comparable accuracy to cycloplegic autorefractometry in children with strabismus. These findings suggest that Mohindra retinoscopy may be used effectively without the need for cycloplegic intervention during follow-up visits, though further research is warranted to confirm its applicability.

## Introduction

Uncorrected or undercorrected refractive errors are the most common cause of vision impairment. According to the World Health Organization (WHO) this problem concerns 153 million people worldwide [1]. It is also considered one of the leading causes of amblyopia [1,2]. Uncorrected refractive errors may also result in lost educational and subsequently employment opportunities and reduced quality of life. Therefore, early detection of refractive errors and their correction may significantly influence someones’ life. Optimal correction of refractive errors appears to be particularly important in children diagnosed with strabismus, which may be caused by, among other things, an associated refractive error [3]. Taken together, measurement of refractive state should be the basis for early childhood vision screening.

The refractive error in young children needs methods, which strongly control status of accommodation. Younger patients present high accommodative reserves, that involve blur-driven accommodation and/or proximal accommodation when refractive status is measured. It may result in refractive bias that produces myopic over-correction. As a consequence, cycloplegic refraction is considered as the gold standard for the investigation of refractive errors in patients with active accommodation, i.e., children, but also young adults [4]. Cycloplegic refraction includes two techniques: cycloplegic autofractometry and wet retinoscopy (cycloplegic retinoscopy). Although studies revealed no or small differences in spherical equivalent or cylinder power determined using these two methods, it should be noted that, even observed, they were clinically insignificant, since the mean differences were smaller than 0.25 diopters (D) [1,5].

Although using cycloplegic drugs (1% atropine or 1% cyclopentolate in case of young children) effectively eliminate accommodation, the discomfort of instilling the drops may make the child uncooperative and further measurement impossible. In some cases, the results obtained by cycloplegic autofractometry may be doubtful, especially in young (under 2 years), or developmentally delayed children, or in patients with nystagmus since autofractometry measurements needs stable fixation for some period of time. In case of older children, the need for the re-visit for postcycloplegic examination of refraction to make a final decision related to prescribed power is also very common. It is also important to note that the use of cycloplegia means that many of the tests to assess visual function, which require good accommodation and a narrow pupil, cannot be performed afterwards. As a result, many of the functional tests cannot be performed at the visit where cycloplegia is used. Therefore, it is important to look for methods of measuring refraction in children that are highly accurate, but do not rely on measurement alone to change accommodative status over time. Mohindra retinoscopy appears to be such an effective testing method. Moreover, the usage of cycloplegic drugs may be associated with various side effects, e.g., photofobia, flush and fever, tachycardia (more common side effects of atropine), and drowsiness (main side effect of cyclopentolate) [6,7].

Mohindra retinoscopy, known as near retinoscopy, is an alternative technique used for the assessment of refractive status in children, which is the type of non-cycloplegic refraction [8]. Originally, Mohindra retinoscopy is performed on axis at the working distance of 50 cm in a dark room and requires the patient to fixate on the light from the retinoscope with one eye, while the other is occluded [8] Although, Wesson et al. confirmed that there is no substantial difference in the result if binocular fixation is allowed [9], especially on patients with strabismus, Mohindra should be performed monocularly to ensure the fixation by the strabismic eye (see Methods section). Under such conditions, where the retinoscope light serves as the fixation target and the room remains dark, the measured refractive state depends solely on the refractive error and tonic accommodation [10]. Originally, Mohindra recommended subtracting a working distance adjustment of 1.25D (-2.00 from the working distance and +0.75 accommodative tonus) from the gross value of the power to obtain the net finding. However, tonic accommodation is greater in infants and less in adults, so Saunders and Westall suggested the use of a modified working distance adjustment even in older children [11]. According to their research, subtraction of 0.75 D from the gross retinoscopy result in children under 2 years and of 1.0 D in children over 2 years and, 1.25 D in adults is recommended [11]. Therefore, the subtracted value takes into account the working distance (50 cm = -2.00 D) and the residual accommodation in the absence of stimuli, which is age dependent (i.e., + 1.25 D in children under 2 years, + 1.00 D in older children and +0.75 D in adults). Near retinoscopy is recommended by American Optometric Association, when a child or parent is anxious about instillation of cycloplegic agents, or the child has had, or is at risk for adverse effects to cycloplegic agents [12].

Studies which previously examined the reliability of Mohindra retinoscopy compared the refractive outcomes with retinoscopy under the cycloplegia [13–15]. Borghi and Rouse [13] showed that retinoscopy under cycloplegia revealed more hyperopia (+0.50 to +0.75 D) than Mohindra retinoscopy. On the other hand, Kauser et al. [14] indicated that Mohindra retinoscopy underestimates myopia and overestimates hypermetropia by 0.3 D. Morales Ruiz et al. [15] revealed strong correlation between Mohindra retinoscopy and cycloplegic retinoscopy, however they observed more significant differences between the outcomes measured using both techniques in case of larger hyperopia.

As mentioned before, previous studies usually compared the measurement of refractive errors using two types of retinoscopy: dry and wet. Furthermore, most studies compared refractive status in children without strabismus. So far, specialists doubt whether this method is accurate in children with large refractive errors and the unstable vision that occurs in strabismus. As the measurement of refractive error with autorefractometers became more and more popular among eye specialists, in the present study we were especially interested in testing the agreement between refractive parameters measured with Mohindra retinoscopy and cycloplegic autorefractometry in children diagnosed with strabismus.

## Methods

### Participants

A total of 43 strabismic children were selected for analysis from the 70 children with suspected binocular vision disorders who underwent eye examinations in this project (26 children excluded from further study had no strabismus and 1 had no refractive error). They were 18 males and 25 females. Mean age was 5.5 years old (range: 3–10 years). Inclusion criteria were: horizontal strabismus (esotropia or exotropia) present with and without the correction of the refractive error (non-refractive or partially refractive heterotropia) and refractive error higher than +0.50 D. 6 participants demonstrated intermittent exotropia and 37 of subjects were diagnosed with constant esotropia measured with the prismatic cover test. 17 (40%) of the participants were wearing glasses (habitual correction) before they participated in the study, which corrected their refractive error only partially. None of the subjects wore a correction that compensated for their total refractive error. In their habitual correction, the subjects were undercorrected relative to both retinoscopy and cycloplegic autorefractometry. Detailed visual parameters of the subjects are presented in Table 1.

**Table 1:** Characteristic of the subjects. Magnitude of ocular misalignment with the correction of the refractive error was measured with prismatic cover test method.

Subject	Age [years]	Sex	FAR TROPIA [PD]	NEAR TROPIA [PD]
s1	5	M	4 ET OD	15 ET OD
s2	6	F	20 AET	20 AET
s3	7	F	12 ET OD	25 ET OD
s4	8	M	12 ET OD	12 ET OD
s5	7	F	20 ET OS	20 ET OS
s6	3	M	20 ET OD	25 ET OD
s7	3	F	45 AET	50 AET
s8	3	F	40 AXT	40 AXT
s9	7,5	M	12 ET OS	14 ET OS
s10	6	M	12 ET OD	30 ET OD
s11	3,5	M	35 ET OD	40 ET OD
s12	10	M	30 ET OS	35 ET OS
s13	7	F	16 ET OD	18 ET OD
s14	6	M	18 ET OD	20 ET OD
s15	3,5	M	25 XT OD	30 XT OD
s16	3	M	30 ET OD	30 ET OD
s17	10	F	4 AET	16 AET
s18	8	M	20 ET OS	25 ET OS
s19	6	M	30 ET OS	35 ET OS
s20	5	F	23 ET OS	25 ET OS
s21	4	M	35 AET	30 AET
s22	7	F	15 AET	15 AET
s23	6	F	20 AET	30 AET
s24	5	F	10 AET	12 AET
s25	7	M	5 AET	30 AET
s26	3	F	15 ET OD	18 ET OD
s27	7	F	16 ET OD	25 ET OD
s28	6	F	20 ET OD	20 ET OD
s29	3,5	M	20 AET	22 AET
s30	4,5	M	15 ET OS	18 ET OS
s31	4,5	F	12 ET OS	12 ET OS
s32	6	F	6 ET OD	18 ET OD
s33	4	F	40 AXT	35 AXT
s34	6	M	8 ET OD	10 ET OD
s35	3,5	F	30 XT OD	20 XT OD
s36	5,5	F	30 XT OS	20 XT OS
s37	5	F	6 ET OD	10 ET OD
s38	5,5	F	6 ET OS	30 ET OS
s39	4	F	8 ET OD	20 ET OD
s40	7,5	M	2 AET	12 AET
s41	4	F	20 XT OD	10 AXT
s42	5	F	6 ET OD	10 ET OD
s43	4	F	30 AET	45 AET

M - male; F - female; ET - esotropia; XT - exotropia; OD - right eye; OS - left eye; AET - alternating esotropia; AXT - alternating exotropia.

The recruitment period was from the 5th of May 2017 to the 15th of August 2018.

### Procedure

The study was approved by Poznan University of Medical Sciences Bioethics Committee and was performed in accordance with the Declaration of Helsinki. All subjects were treated in accordance with the recommendations of the Bioethics Committee and each participant had the right to withdraw from the study at any time. Written informed consent was obtained from parents or guardians.

First, the refractive status of each child was assessed by an experienced optometrist (authors of the manuscript: APK or ABK) using non-cycloplegic Mohindra retinoscopy technique. The measurement was performed monocularly (with one eye occluded) in a dark room with the child fixating on the light of the streak retinoscope (Heine BETA 200) at the distance of 50 cm. A working distance of 50 cm from the retinoscope was maintained using a fixed string attached to the retinoscope. Retinoscopy was conducted monocularly (according to the technique originally proposed by Mohindra [8]) to ensure central fixation of the strabismic eye and to measure central, but not peripheral refraction. The aim of the procedure was to determine the powers of the principal meridians using retinoscopy racks or loose lenses in cases of older and younger children, respectively. First of all, the most plus meridian was neutralized which was a sphere meridian, then the retinoscope beam was turned 90 degrees to neutralize the perpendicular meridian. The difference in refractive power of the two principal meridians indicates the amount of astigmatism. As the participants of presented study was over 2 years, a correction factor of -1,00 D was subsequently subtracted from the neutralizing lenses (see: introduction and Saunders and Westall [11]). The obtained refractive powers were then put into trial frames and then the angle of the strabismus was measured with prismatic cover test at distance and at near after 30 minutes of adaptation. The values of eyes deviation are presented in Table 1.

After optometric examination, the patient was referred for an ophthalmological examination, where the anterior and posterior segments of the eyes were assessed and an autorefractometer refraction measurement was performed after the application of cyclopentolate drops. To achieve accommodation paralysis, two drops of 1% cyclopentolate were instilled in the conjunctival cul-de-sac, five minutes apart and the examination of refractive error with a hand-held autorefractometer (Retinomax, model K-plus 3, Righton) was performed 30 minutes after the administration of the last drop. The previous results of the refractive error measurements were not known to any of the researchers. The results were collected and recorded after the research was completed. They were then subjected to statistical analysis.

### Statistical analyses

In order to compare the results of the refractive techniques the following refractive parameters were statistically analyzed: spherical component of refractive error, spherical equivalent of refractive error (SE = S + C/2) and cylinder value. The powers obtained in sphero-cylindrical form were converted to their vector components according to Thibos and Horner [16,17]. Power vectors for astigmatism (J0 Horizontal Jackson-Cross and J45 oblique Jackson-Cross) were calculated to facilitate statistical analysis by transforming astigmatism into a linear coordinate system. These vectors were calculated using the following equations: J0 = -(C/2) cos (2θ) and J45 = -(C/2) sin (2θ), where C is the cylinder power and θ is the cylinder axis in degrees. The Wilcoxon test was used to compare these values, as the data did not follow a normal distribution (as determined by the Shapiro-Wilk test; P < .05), except for J45, which showed a normal distribution (P = .287 and P = .577 for Mohindra retinoscopy and cycloplegic autorefractometry, respectively) and was analyzed using the Student’s t-test. The Bland-Atman analysis and a the two-way random-effects model for absolute agreement with single measures, represented by the Intraclass Correlation Coefficient (ICC were used to check the agreement between the two methods. Additionally, the correlations were tested using the Spearman correlation method and these data are presented as a supplementary analysis. All statistical analyses were conducted using Statistica software version 13.3, except for the ICC analysis, which was performed in PQStat software version 1.8.6.102. Statistical significance was taken as *P* ≤ .05.

## Results

Mean, median of the spherical refractive error, spherical refractive equivalent, cylinder value and cylinder axis and its standard deviation values (SD) and interquartile range (IQR) are presented in the Table 2.

**Table 2:** Mean, median, SD and IQR values obtained with Mohindra retinoscopy and cycloplegic autorefractometry.

Method	Spherical component of refractive error [D] Mean; *(SD)* Median; *(IQR)*	Spherical refractive equivalent [D] Mean; *(SD)* Median; *(IQR)*	Cylinder power [D] Mean; *(SD)* Median; *(IQR)*	J0 [D] Mean; *(SD)* Median; *(IQR)*	J45 [D] Mean; *(SD)* Median; *(IQR)*
mRET	+4.86; *(2.31) *+4.38; *(+3.00 ÷ +6.50)*	+4.57; *(2.21)* +4.25; *(+3.00 ÷ +6.0)*	-0.61; *(0.66)* -0.50; *(-1.00 ÷ 0.00)*	0.19; *(0.48)* 0.11; *(-0.13 ÷ 0.47)*	<0.01; *(0.27)* <0.01; *(-0.21 ÷ 0.22)*
cAR	+4.83; *(2.32)* +4.50; *(+3.00 ÷ +6.50)*	+4.50; *(2.19)* +4.25; *(+2.88 ÷ +6.00)*	-0.66; *(0.68)* -0.50; *(-1.00 ÷ 0.00)*	0.19; *(0.48)* 0.12; (*-0.15 ÷* +0.46)	<0.01; *(0.33)* <0.01; *(-0.23 ÷ 0.20)*

mRET - Mohindra retinoscopy; cAR - cycloplegic autorefractometry; SD - standard deviation; IQR – interquartile range

The range of spherical component of refractive error was + 0.75 to +10.75 D when measured with Mohindra retinoscopy and +1.00 to +10.75 when cycloplegic autorefractometry was used. The median spherical component of refractive error was almost equal for both the methods used (+4.38 D (IQR: + 3.00 to +6.50 D) vs. + 4.50 D (IQR: + 3.00 to +6.50 D), for Mohindra retinoscopy and cycloplegic autorefractometry, respectively; *Z* = 0.61, *P* = .54). The range of spherical equivalent of refractive error was + 0.75 to +10.25 D when measured with Mohindra retinoscopy and +0.88 to +10.25 D when measured with cycloplegic autorefractometry. Median spherical refractive equivalent did not differ between two methods (+4.25 D (IQR: from +3.00 to +6.00 D) vs. + 4.25 D (IQR: from +2.88 to +6.00 D) for Mohindra retinoscopy and cycloplegic autorefractometry, respectively; *Z* = 0.89, *P* = .37).

**Fig 1:**
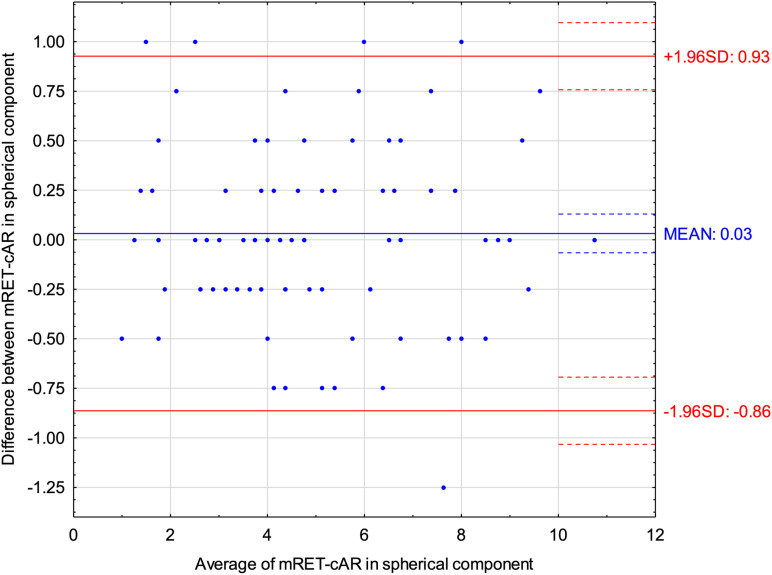
Bland-Altman plot displaying the differences for spherical component of refractive error between Mohindra retinoscopy (mRET) and cycloplegic autorefractometry (cAR).

**Fig 2:**
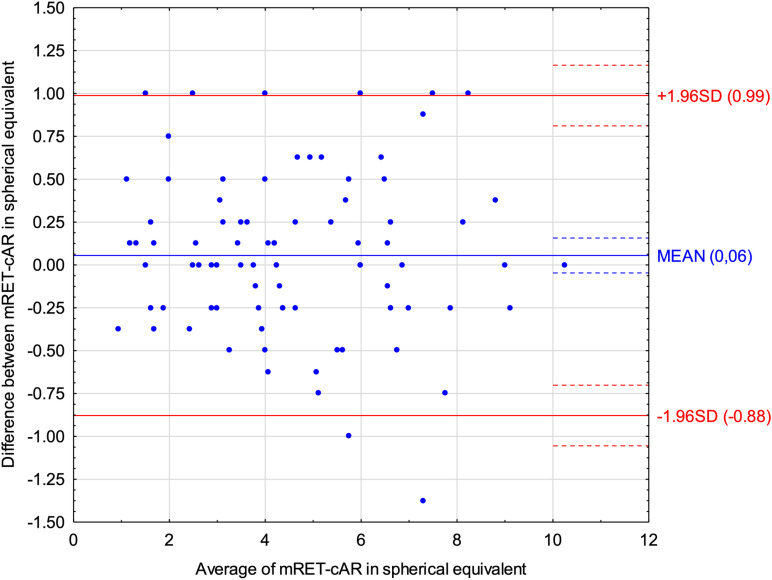
Bland-Altman plot displaying the differences for spherical equivalent of refractive error between Mohindra retinoscopy (mRET) and cycloplegic autorefractometry (cAR).

Bland-Altman plots for the spherical component of refractive error (Fig 1) revealed that Mohindra retinoscopy indicated on average 0.03 D more positive results than cycloplegic autorefractometry. For the spherical equivalent of refractive error (Fig 2) Mohindra retinoscopy showed 0.06 D more positive results than cycloplegic autorefractometry. An ICC of 0.98 (95% CI: 0.97–0.99) and 0.98 (95% CI: 0.96–0.98) was found for spherical component and spherical equivalent of refractive error respectively, indicating excellent reliability between methods. Table 3. presents additionally the data from the Bland-Altman analysis for spherical component and equivalent of refractive error, cylinder power, and J0 and J45 vectors, including the mean difference, standard deviation of the difference, and its lower and upper limits of agreements.

**Table 3:** Bland-Altman analysis results (mean difference, standard deviation of the difference, lower and upper limits of agreement) and interclass correlation coefficient (ICC).

Parameter	Mean Difference [D]	Standard Deviation of Difference [D]	Lower Limit of Agreement [D]	Upper Limit of Agreement [D]	ICC (95%CI)
Spherical Component	0.03	0.46	-0.86	0.93	0.98 (0.97–0.99)
Spherical Equivalent	0.06	0.48	-0.88	0.99	0.98 (0.96–0.98)
Cylinder Power	0.05	0.35	-0.63	0.73	0.86 (0.80–0.91)
J0 Vector	< 0.01	0.16	-0.31	0.32	0.94 (0.90–0.97)
J45 Vector	< 0.01	0.15	-0.29	0.29	0.88 (0.80–0.93)

Astigmatism was detected in 50 eyes. Astigmatism was considered when it was detected by both methods and had a value of ≥ 0.25 D. The cylinder power measured using Mohindra retinoscopy ranged up to 2.50 D, while using cycloplegic autorefractometry up to 3.00 D. Mean and median power for the cylinder, as well as power vectors for astigmatism J0 and J45, obtained with Mohindra retinoscopy and cycloplegic autorefractometry are presented in the Table 2. Median cylinder power was the same when measured with Mohindra retinoscopy and with the cycloplegic autorefractometry (-0.50 D (IQR: from -1.00 to 0.00 D) for Mohindra retinoscopy and cycloplegic autorefractometry; *Z* = 1.15, *P* = .25). No differences were also found for J0 (0.11 D (IQR: from -0.13 to 0.47 D) vs. 0.12 D (IQR: from -0.15 to +0.46 D) for Mohindra retinoscopy and cycloplegic autorefractometry, respectively; *Z* = 0.12; *P* = .91) and for J45 vectors (< 0.01 D (SD 0.27) vs. < 0.01 D (SD 0.33) for Mohindra retinoscopy and cycloplegic autorefractometry, respectively; t(49) = -0.16, *P* = .88).

**Fig 3:**
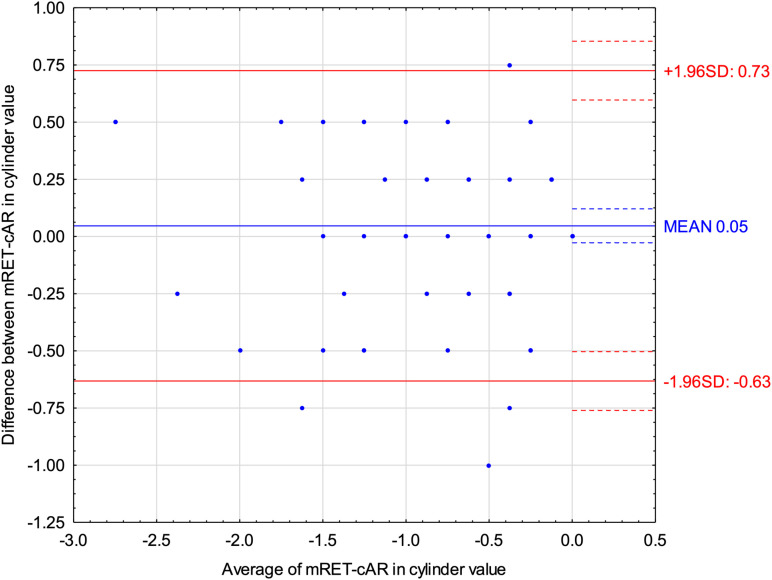
Bland-Altman plot displaying the differences for cylinder value between Mohindra retinoscopy (mRET) and cycloplegic autorefractometry (cAR).

Bland-Altman plots for the cylinder value (Fig 3) revealed that Mohindra retinoscopy indicated on average 0.05 D more positive results than cycloplegic autorefractometry. ICC for cylinder power was 0.86 (95% CI: 0.80–0.91), indicating good reliability between the two methods.

**Fig 4:**
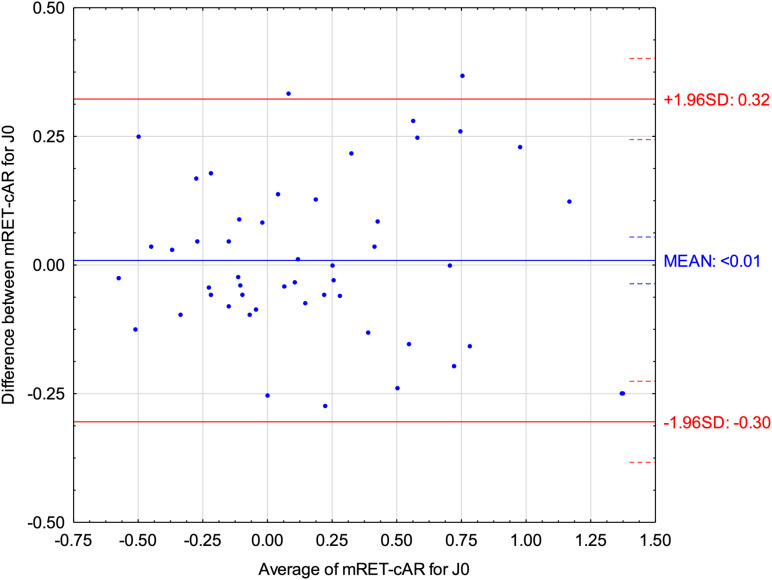
Bland-Altman plot displaying the differences for J0 vector between Mohindra retinoscopy (mRET) and cycloplegic autorefractometry (cAR).

**Fig 5:**
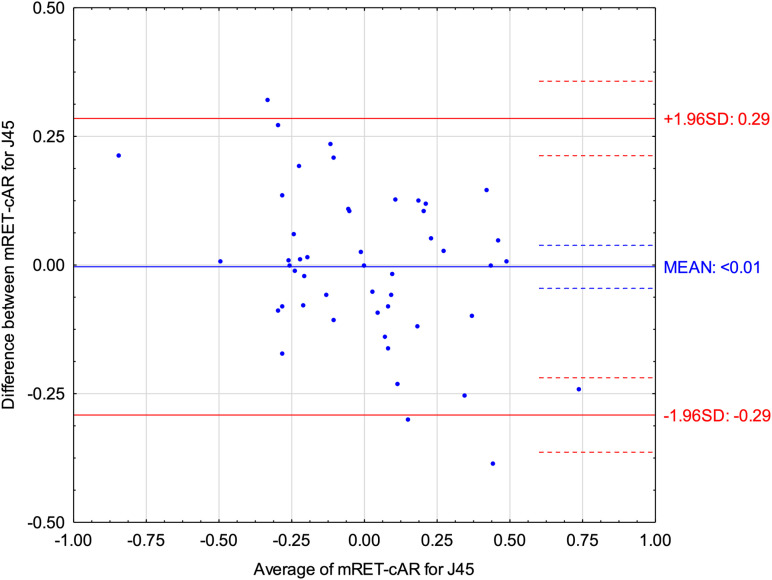
Bland-Altman plot displaying the differences for J45 vector between Mohindra retinoscopy (mRET) and cycloplegic autorefractometry (cAR).

Figs 4 and 5 show Bland–Altman plots of refractive components J0 vector and J45 vector comparing Mohindra retinoscopy with cycloplegic autorefractometry. Both indicated small differences between both methods (< 0.01). ICC for J0 vector was 0.94 (95% CI: 0.90–0.97) and for J45 was 0.88 (95% CI: 0.80–0.93).

Additional correlation analyses between the parameters obtained by the two methods can be found in the supplementary materials.

## Discussion

The purpose of this study was to compare the refractive error results obtained with Mohindra retinoscopy and cycloplegic autorefraction in children with strabismus. The question of whether cycloplegic refraction is always necessary in children, even in those with strabismus, may be answered by such a comparison.

The results revealed that the median spherical component of refractive error was almost equal for both methods used (+4.38 D vs. + 4.50 D, for Mohindra retinoscopy and cycloplegic autorefractometry, respectively; *P* = .54). Similar findings concern median spherical refractive equivalent (+4.25 for both methods; *P* = .29). Interestingly, the Bland-Altman analysis revealed that the mean differences in the spherical component and spherical equivalent of refractive errors between the two methods were less than 0.1 D. Overall, Mohindra retinoscopy tended to yield slightly more hyperopic measurements. Median cylinder powers were the same for both methods (-0.50 D; *P* = .25). No differences were also found for J0 (*P* = .91) and J45 vectors (*P* = .88) between Mohindra retinoscopy and cycloplegic autorefractometry.

To the best of our knowledge, no study has compared Mohindra retinoscopy with cycloplegic autorefractometry especially in strabismic subjects. Cycloplegic autorefraction has been commonly compared with cycloplegic retinoscopy. The study by Guha et al. [18] showed good agreement of cycloplegic autorefraction with retinoscopy under cycloplegia in identifying all types of the refractive errors. However, they noted that clinically significant differences were more likely to occur in children less than six years of age. Furthermore, it seems that the type of autorefractometer (hand-held or table-mounted), does not affect the results when used under cycloplegia, as it does under non-cycloplegic conditions [19]. On the other hand, the study by Kurtent [20], which compared cycloplegic hand-held and table-mounted autorefraction with cycloplegic retinoscopy revealed that hand-held autorefractor recording provided lower measurements compared to the other two methods. It was particularly noticeable in higher sphere and cylinder values. These different outcomes may be a result of different autorefractometers used in both studies. Although the study by Kurtent [20] also included children with strabismus, it did not analyze strabismic participants as a separate group, so it is not possible to conclude whether the results obtained differ between strabismic and non-strabismic patients.

Few studies have focused on the comparison of Mohindra retinoscopy and cycloplegic retinoscopy in children. To avoid differences related to the topical agent used, the studies presented applied 1% cyclopentolate, which is recommended by The American Optometric Association in children older than one year old to avoid undesired side effects [7]. One of the first studies was carried out in early primary grade schoolchildren by Mohindra and Molinari [21] and revealed that both techniques provide similar objective measurements of refraction. Similar conclusions were reached in the study by Morales Ruiz et al. [15] conducted recently and in the study on children diagnosed with Down syndrome [22]. Borghi and Rouse [13] showed that cycloplegic retinoscopy gave 0.50 to 0.75 D more plus than Mohindra retinoscopy, whereas Saunders and Westall [11] indicated that the difference between both methods was 0.39 D (more plus for cycloplegic retinoscopy). The differences between both methods obtained in these studies may stem from the fact that in the study by Borghi and Rouse [13] the value of 1.25 D was subtracted from the sphere power, as indicated initially by Mohindra. As the following study by Saunders and Westall [11] suggested, the subtraction of 1.00 D (in case of children >2 years) or 0.75 D (in case of children <2 years) improves the agreement between two techniques. Our study, which demonstrated a high agreement between the two techniques used, suggests that subtracting 1.00 D (assuming that children have greater tonic accommodation, i.e., 1.00 D instead of 0.75 D, especially hyperopic children) in children over 3 years of age (as was the case in our study) is a more appropriate approach and supports the suggestion made by Saunders and Westall [11]. Interestingly, Mohindra retinoscopy was also performed in children diagnosed with accommodative esotropia to test whether this method could be used to determine optical correction [23]. Although the correlation between Mohindra retinoscopy and cycloplegic retinoscopy was good, the authors recommended that Mohindra retinoscopy should be used only as a screening method for refractive errors. Saunders and Westall [11] revealed that Mohindra retinoscopy can be used successfully in children younger than 2 years, however some studies have shown that Mohindra retinoscopy does not give reliable results compared to cycloplegic refraction [9,24]. Twelker and Mutti [24] revealed that in case of infants (children younger than 12 months), Mohindra retinoscopy underestimated hyperopia by approximately 1.0 D compared to cycloplegic retinoscopy with 1% cyclopentolate, whereas Wesson et al. [9] showed that the difference between the two methods was even higher, reaching the value of 2.12 D. Wesson et al. [9] also studied older children and noticed that the agreement between cycloplegic retinoscopy and Mohindra retinoscopy increased with the age of the patient. A similar observation was reported in the study by Mutti et al. [25] who showed that the agreement between cycloplegic retinoscopy and Mohindra retinoscopy was lower in children at 3 months, which then improved at 9 and 18 months (which was still quite modest).

Based on the results presented above, the question arises - how can these discrepancies in agreement between the two methods reported previously and in the present study be explained? First, our study includes children between the ages of 3 and 10 years, which may have a positive impact on the excellent correlation. Second, we can also consider the factor of experience. The proficiency and experience of the examiners are important factors affecting the accuracy of retinoscopy [26]. Retinoscopy procedure is subject to measurement bias and intra- and inter-observer variability [27]. Nevertheless, previous studies indicated that experienced eye specialists can obtain accurate measurements [28,29]. In our study, the Mohindra retinoscopy technique was performed by highly experienced optometrists (experience of more than 10 years), which may have contributed to the high correlation between the results of the two techniques. It can therefore be concluded that the Mohindra retinoscopy method is an effective technique, especially well-trained specialists who have had many hours of retinoscopy training and, after several years of practice, can usually assess the refractive error with a high degree of accuracy.

Moreover, according to many opinions, hyperopia is problematic refractive error that can only be properly measured under cycloplegia to effectively eliminate the accommodative process [30,31]. It is thought that cycloplegic refraction is a crucial procedure in the evaluation of strabismus, especially in accommodative esotropia [32]. However, our study shows that it is possible to detect refractive error with Mohindra retinoscopy in hyperopic strabismic children without paralysis of accommodation. Nonetheless, it should be noted that about 40% of the children in our study were already using some form of ocular correction, i.e., they had partially relaxed accommodation. This shows that the Mohindra retinoscopy method works well during follow-up visits, but it is advisable to use cycloplegic drops for the initial assessment of refractive error, so that high hyperopia is not overlooked, especially in strabismic children.The last point to consider is that, although our results indicate a high agreement between the Mohindra retinoscopy and cycloplegic autorefraction, this high agreement is composed of Mohindra-derived measurements, some of which show both underestimation and overestimation of refractive values compared to cycloplegic autorefraction. Although this suggests that the method is not biased toward either the plus or minus direction (no myopic or hyperopic trend), considering the absolute values of these differences reveals a measurement discrepancy of approximately 0.5 D. While this difference may not be statistically significant, it could still be clinically relevant in certain cases. As our study revealed, the difference between the two methods can range from as little as 0 to as much as 1.00 D. While a variation of ±0.25 D is generally considered within acceptable measurement error, a discrepancy of 1.00 D is clinically significant and can meaningfully affect the final prescription.

### Limitations of the study and future direction

Although the results of the studies presented look promising, it’s important to note that the number of participants involved in our study is not large. It would be necessary to carry out studies on hundreds of children with strabismus in order to establish whether the method is really effective and can be transferred to the general population. Subsequent trials would need to include up to hundreds of children of different ages. It would also be interesting to see to what extent the refractive results obtained with Mohindra retinoscopy differ from those obtained with cycloplegic autorefractometry when the measurements are performed by practitioners with little experience in retinoscopy. Finally, it is important for future studies to investigate whether Mohindra retinoscopy is also accurate in children with myopia and to assess the potential impact of clinician experience on the results.

### Conclusions

In conclusion, our study showed that Mohindra retinoscopy can be a valuable method for the detection of refractive errors in strabismic children over the age of 3 years, as we reported good agreement between spherical component, spherical equivalent and astigmatic vectors of refractive errors obtained by Mohindra retinoscopy and cycloplegic autorefractometry. This suggests that both methods can be considered interchangeable, especially during the follow-up visits. This method can also be used in situations where the administration of cycloplegic drops cannot be used, e.g., for health reasons, or when examinations are carried out outside medical clinics (e.g., in schools, care centers, etc.). The absence of cycloplegia for follow-up visits also allows functional tests to be performed in a single visit, such as assessment of accommodation, vergence, binocular vision and others that cannot be performed after paralysis of accommodation. However, this method is useful especially in the hands of experienced practitioners.

## Supporting information

S1 Fig Correlation of spherical component of refractive error measured with Mohindra retinoscopy (mRET) and cycloplegic autorefractometry (cAR). The Spearman correlation revealed a very strong positive correlation for the spherical component (S1 Fig) and the spherical equivalent of refractive error (S2 Fig) measured using Mohindra retinoscopy and cycloplegic autorefractometry (spherical component: r² = 0.98, P < .001; spherical equivalent: r² = 0.98, P < .001). Similarly, a strong correlation was observed for the cylinder value (r² = 0.80, P < .001; S3 Fig).
(TIF)

S2 Fig Correlation of spherical equivalent of refractive error measured with Mohindra retinoscopy (mRET) and cycloplegic autorefractometry (cAR). The Spearman correlation revealed a very strong positive correlation for the spherical component (S1 Fig) and the spherical equivalent of refractive error (S2 Fig) measured using Mohindra retinoscopy and cycloplegic autorefractometry (spherical component: r² = 0.98, P < .001; spherical equivalent: r² = 0.98, P < .001). Similarly, a strong correlation was observed for the cylinder value (r² = 0.80, P < .001; S3 Fig).
(TIF)

S3 Fig Correlation of cylinder value measured with Mohindra retinoscopy (mRET) and cycloplegic autorefractometry (cAR). The Spearman correlation revealed a very strong positive correlation for the spherical component (S1 Fig) and the spherical equivalent of refractive error (S2 Fig) measured using Mohindra retinoscopy and cycloplegic autorefractometry (spherical component: r² = 0.98, P < .001; spherical equivalent: r² = 0.98, P < .001). Similarly, a strong correlation was observed for the cylinder value (r² = 0.80, P < .001; S3 Fig).
(TIF)
