# Supplementary figures and images for: Accuracy of refractive error measurements in children with strabismus comparing cycloplegic autorefractometry to dry monocular Mohindra retinoscopy
Source: PLoS One. 2025 Jun 2;20(5):e0323750. doi: 10.1371/journal.pone.0323750 (PMC12129147; doi:10.1371/journal.pone.0323750)

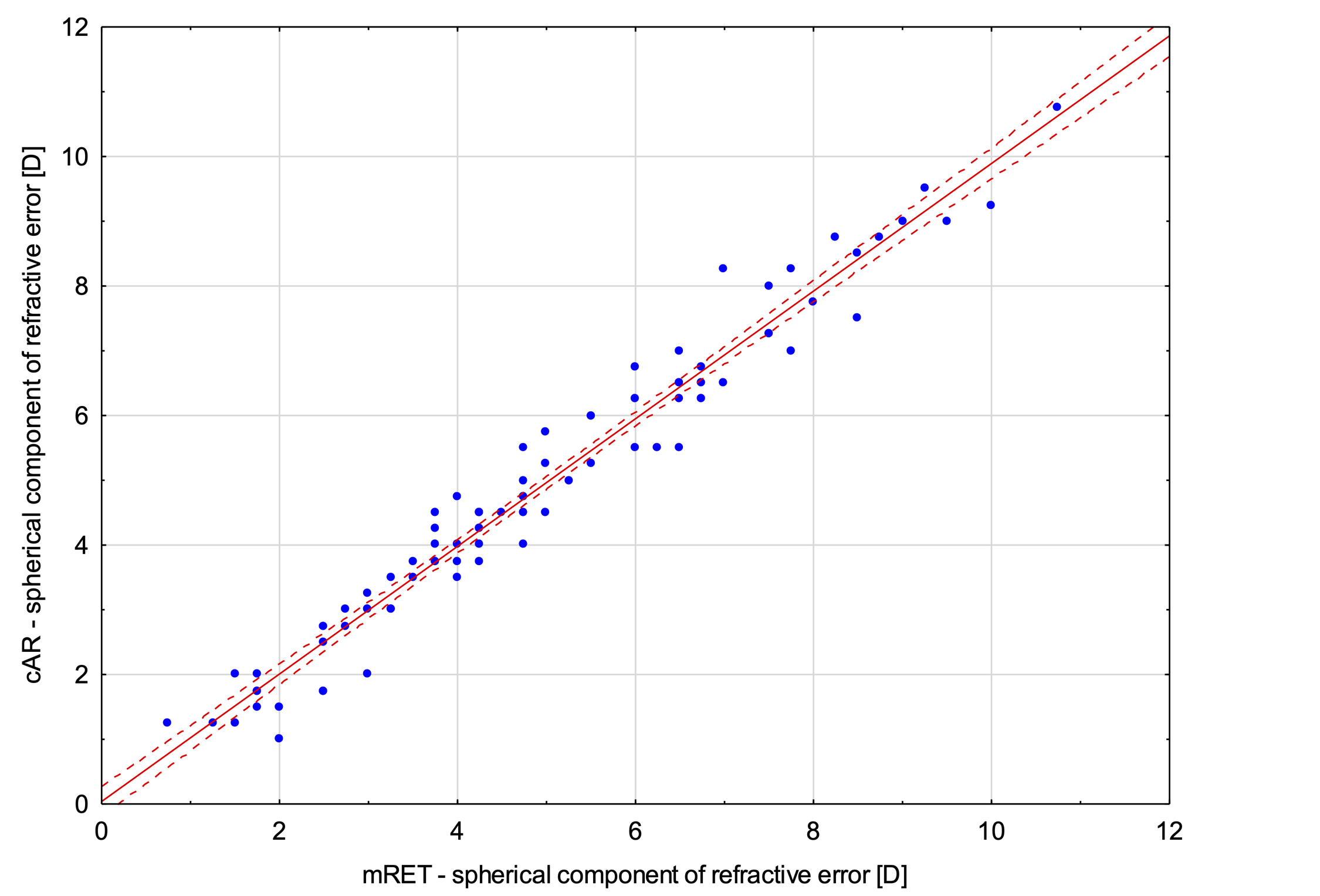

Supplement: S1 Fig — The Spearman correlation revealed a very strong positive correlation for the spherical component (S1 Fig) and the spherical equivalent of refractive error (S2 Fig) measured using Mohindra retinoscopy and cycloplegic autorefractometry (spherical component: r² = 0.98, P < .001; spherical equivalent: r² = 0.98, P < .001). Similarly, a strong correlation was observed for the cylinder value (r² = 0.80, P < .001; S3 Fig). (TIF) [file pone.0323750.s001.tif]

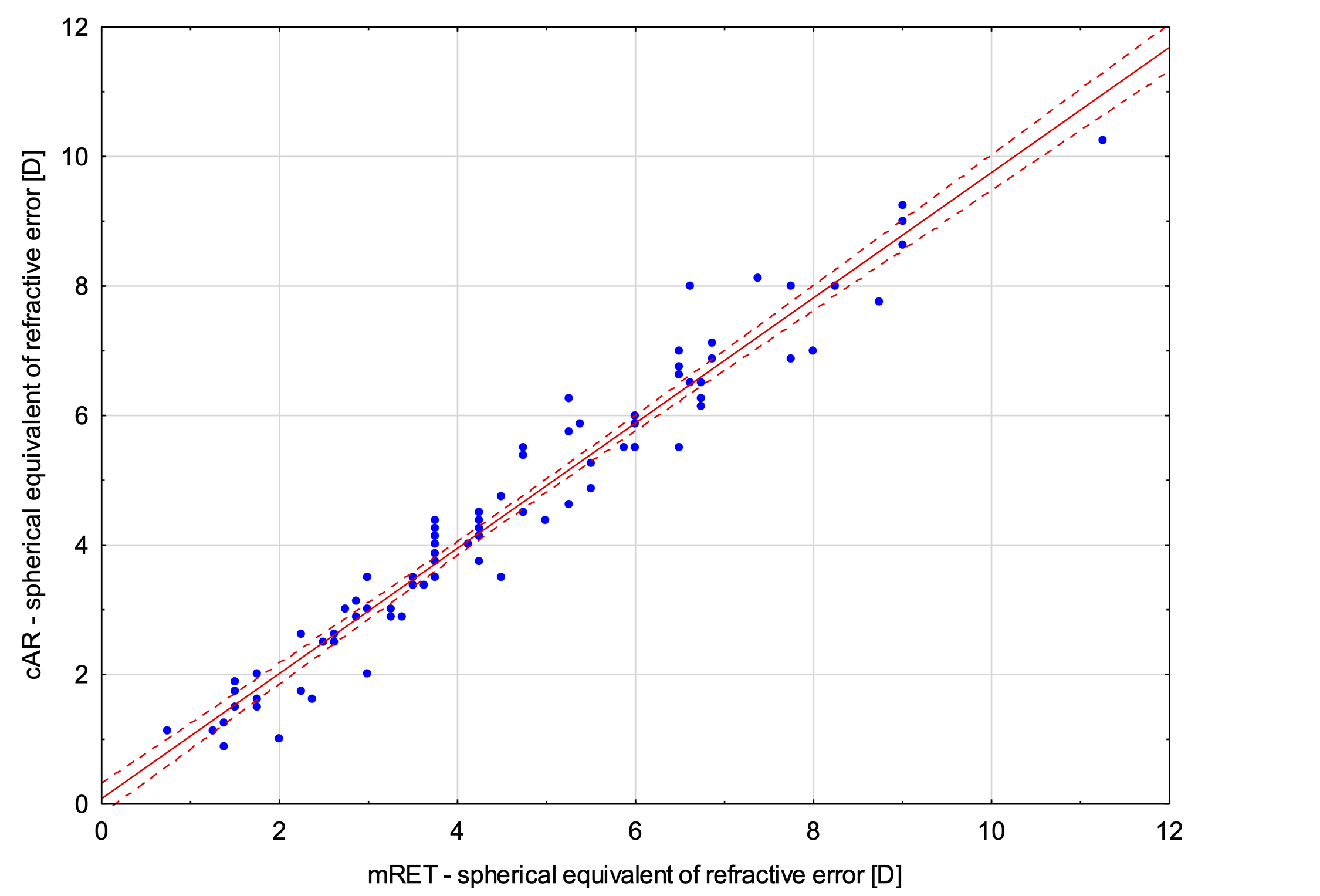

Supplement: S2 Fig — The Spearman correlation revealed a very strong positive correlation for the spherical component (S1 Fig) and the spherical equivalent of refractive error (S2 Fig) measured using Mohindra retinoscopy and cycloplegic autorefractometry (spherical component: r² = 0.98, P < .001; spherical equivalent: r² = 0.98, P < .001). Similarly, a strong correlation was observed for the cylinder value (r² = 0.80, P < .001; S3 Fig). (TIF) [file pone.0323750.s002.tif]

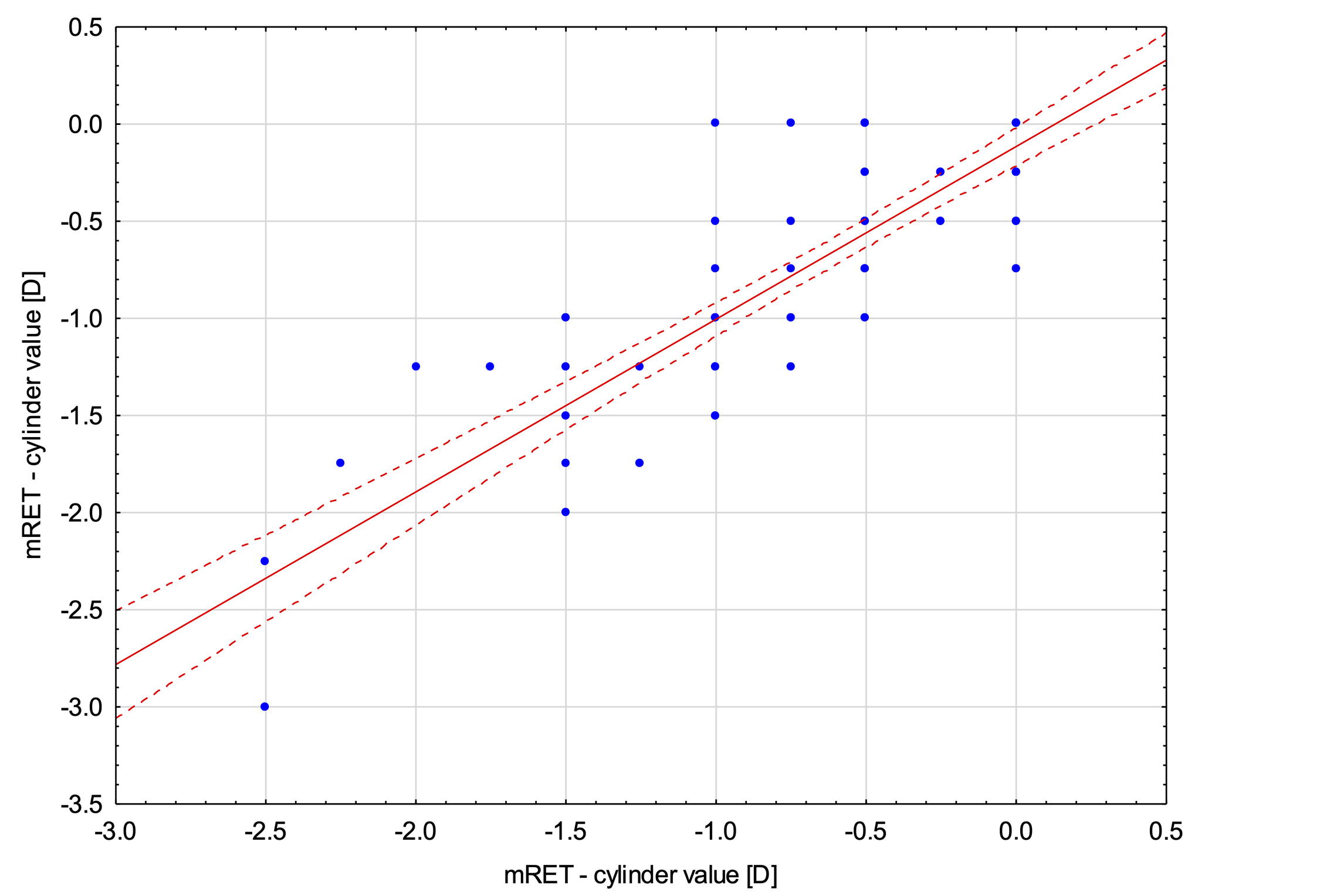

Supplement: S3 Fig — The Spearman correlation revealed a very strong positive correlation for the spherical component (S1 Fig) and the spherical equivalent of refractive error (S2 Fig) measured using Mohindra retinoscopy and cycloplegic autorefractometry (spherical component: r² = 0.98, P < .001; spherical equivalent: r² = 0.98, P < .001). Similarly, a strong correlation was observed for the cylinder value (r² = 0.80, P < .001; S3 Fig). (TIF) [file pone.0323750.s003.tif]
